# Half-century trends in alpha and beta diversity of phytoplankton summer communities in the Helsinki Archipelago, the Baltic Sea

**DOI:** 10.1093/plankt/fbac029

**Published:** 2022-06-12

**Authors:** Kalle Olli, Emil Nyman, Timo Tamminen

**Affiliations:** Centre for Limnology, Institute of Agricultural and Environmental Sciences, Estonian University of Life Sciences, Kreutzwaldi 5, Tartu 51006, Estonia; Institute of Ecology and Earth Sciences, University of Tartu, Liivi 2, 50409, Estonia; Urban Environment Division, City of Helsinki, Työpajankatu 8, 00580 Helsinki, Finland; Marine Research Centre, Finnish Environment Institute, Latokartanonkaari 11, FI-00790 Helsinki, Finland

**Keywords:** biodiversity trend, beta diversity, beta decomposition, species turnover, eutrophication

## Abstract

We analyzed phytoplankton biodiversity trends in a 52 year (1967–2018) monitoring time-series from the archipelago of Helsinki, Gulf of Finland, the Baltic Sea. The community ordination revealed strong ordering of samples along the time axis (generalized additive model—gam fit: *R*^2^ = 0.9). Species richness increased in time and was the most influential alpha diversity descriptor related to the community structure (gam fit: *R*^2^ = 0.56–0.70). Changes in species richness accounted for 35–36% of the mean between-sample beta diversity. The remaining 64–65% was due to species turnover—the dominant component of the biodiversity trend. The temporal beta diversity trend reflected the eutrophication history of the geographically confined region, with a turning point in mid-1990s demarking the adaptation and recovery phases of the phytoplankton community. Trends in spatial beta diversity revealed homogenization of the communities in the outer archipelago zone, but not in the inner bays. The temporal decay of community similarity revealed high turnover rate, with 23.6 years halving time in the outer archipelago and 11.3 years in the inner bays, revealing the differences in eutrophication strength. The observed phytoplankton trends manifest the regional eutrophication history, and dispersal of new species to the unsaturated brackish species pool.

## INTRODUCTION

Coastal communities provide a range of ecosystem services, like recreation, carbon storage or interception of nutrients flowing from land to the sea (Carstensen *et al*., 2020). Yet in recent decades these services are put at risk or degraded by external pressures (Heckwolf *et al*., 2021). A management-relevant action to mitigate deterioration of ecosystem services and functions is detection of biodiversity change.

Quantifying trends in biodiversity is a non-trivial task, because biodiversity itself is a multifaceted concept, involving genetic, taxonomic, phylogenetic and ecological components. Because it is relatively easy to observe, record and interpret, species richness has remained the dominant metric of biodiversity and its change in natural ecosystem monitoring. Furthermore, the current biodiversity crisis refers most often to decline of global species richness, although as recently documented by several global meta-analyses, little if any net change in local species richness through time has occurred (Dornelas *et al*., 2014; Elahi *et al*., 2015; Li *et al*., 2020; Pilotto *et al*., 2020). It has been argued that not just the number of species, but species turnover—the change in the identity of species and their relative proportions—is more sensitive to signal changes in biodiversity than richness measures (Dornelas *et al*., 2014; Hillebrand *et al*., 2018; Kortz and Magurran, 2019). Beta diversity—the change in species composition per unit space or time—is inherently suited to analyze trends in biodiversity, as it natively compares two or more samples, allowing a flexible variety of metrics (Jost, 2007; Tuomisto, 2010).

Phytoplankton is a key indicator of the state of aquatics ecosystems, as it is essential for carbon and nutrient turnover, and provides a primary energy source for the whole food web (Carvalho *et al*., 2013). The high turnover and sensitivity to external variables makes phytoplankton a sensitive indicator of environmental change in time scales from days and weeks to decades. Despite the far-reaching importance, knowledge on long-term trends in coastal phytoplankton diversity and community composition remains understudied, partly due to the rarity of consistent time-series.

The Baltic Sea is a shallow semi-enclosed estuarine brackish water body (422 × 10^3^ km^2^) in the temperate zone of the northern hemisphere. It is non-tidal and has a long water residence time of >30 years. The Baltic Sea is endangered by large nutrient inputs from the more than four times larger (ca. 2 million km^2^) and highly populated (ca. 85 million people) drainage area, shared by 12 industrialized countries, 9 of which are bordering the coast (Snoeijs-Leijonmalm and Andrén, 2017).

The Baltic Sea has a notorious history of eutrophication, with clear signs from the late 19th century (Finni *et al*., 2001; Andersen *et al*., 2017), and documented summer cyanobacterial blooms from 1850s (reviewed in Olli, 1996). After the WW II, intensifying agriculture in the drainage area enhanced nutrient run-off to the sea (Gren *et al*., 2000). The anthropogenic nutrient loading has left signs of eutrophication in the bottom sediments as elevated organic carbon and cyanobacterial pigment content since the mid-1960s (Poutanen and Nikkilä, 2001). In the 1970s and 1980s, the increasing loads of nitrogen and phosphorus to the Baltic Sea led to accelerated eutrophication and increased phytoplankton biomass (Kuparinen and Tuominen, 2001). A self-sustaining feed-back loop, involving spreading of deep water anoxia and N-fixing cyanobacterial blooms, kept the Baltic Sea in a “vicious circle” of eutrophication despite considerable management efforts (Vahtera *et al*., 2007; Carstensen *et al*., 2014). In recent decades, the direct nutrient inputs to the Baltic Sea have decreased greatly since peaking in the 1980s, and as a consequence nutrient concentrations in the Baltic Sea have decreased, although not as rapidly as nutrient inputs (Heiskanen *et al*., 2019).

It is reasonable to assume that the phytoplankton community composition would respond and adapt to the changing eutrophication levels, yet species-specific, fully quantitative data are not available from the pre-eutrophication period. Variation and long-term trends of the Baltic Sea phytoplankton composition have been analyzed statistically with various methods in the southern (Wasmund *et al*., 1998, 2011; Wasmund and Uhlig, 2003), as well as in the northern Baltic Sea (Suikkanen *et al*., 2007; Jaanus *et al*., 2011). Recently, Griffiths *et al*. (Griffiths *et al*., 2020) found no common trends in Baltic Sea summer phytoplankton biomass from a range of pelagic locations, suggesting that the processes governing biomass build-up have considerable spatial idiosyncrasy.

Previous analyses have demonstrated notable decadal-scale shifts in the phytoplankton community structure in all the major sub-basins of the Baltic Sea (Olli *et al*., 2011, 2013). This suggests that although phytoplankton total biomass follows heterogeneous temporal trajectories in response to local eutrophication pressure, the regional community structure has also common temporal dynamics. Here, we present trends in phytoplankton community structure by using a coherent and exceptionally long (1966–2018) fully quantitative time-series from the Helsinki Archipelago—a spatially confined coastal region in the central Gulf of Finland that has undergone a similar eutrophication history to the rest of the Baltic Sea (Finni *et al*., 2001). Local nutrient loads to the area peaked for phosphorus in the 1970s and for nitrogen as late as in the early to mid-1990s (Vaalgamaa, 2004). Most of the point source nutrient load originating from several wastewater treatment plants affecting the shallow near coastal waters was diverted to the outer archipelago zone from 1987 onwards.

We hypothesized that the previously observed long-term (1966–2008) shifts in Baltic Sea phytoplankton community structure (Olli *et al*., 2011) and increasing trends in species richness (Olli *et al*., 2014) will continue and can be further validated with new monitoring data up to 2018. We tested a range of alpha diversity metrics for their sensitivity to catch the community time trend. Further, we analyzed trends in temporal and spatial beta diversity, which are highly sensitive metrics of biodiversity change in response to environmental pressures (McGill *et al*., 2015; Hillebrand *et al*., 2018). Beta diversity—the change in community composition per unit space or time (Whittaker, 1960)—is a central concept in ecology and informs on the relationships between species and their environment along a spatial, ecological, or temporal gradient. We discussed potential drivers of the community composition, which in the inner shallow bays of the archipelago reflect the trajectories of severe eutrophication and recovery thereafter.

## METHODS

### Source of data

**Fig. 1:**
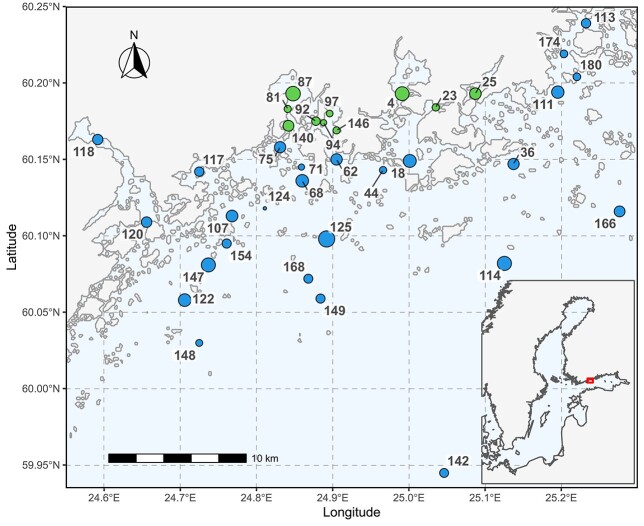
Sampling stations, denoted by numeric codes, in the Helsinki archipelago. The location of the study region in the Baltic Sea is shown with red rectangle in the bottom-right insert figure. Main figure symbol sizes are proportional to the square root of the number of quantitative phytoplankton samples (total = 4630). The green symbols denote stations from the inner bays and the blue symbols the other archipelago stations (see text for distinction criteria).

The 52-year phytoplankton time-series, covering years 1966–2018, originated from the City of Helsinki Urban Environment Division. The data are geographically constrained, with a maximum latitudinal and longitudinal range of 20 and 30 km, respectively (Fig. 1). The monitoring program evolved in time towards lower number of statutory stations and higher resolution in sampling. Annual sampling frequency ranged from 22 to 286 (interquartile range 43–129), and was seasonally well balanced within any particular year.

The study region is a coastal archipelago, with station depths ranging from 2.5 to 71 m, and distance to the closest point on mainland shore from 80 m to 23.6 km. The interquartile range of salinity was 4.9–5.6. The seasonally ice covered northern Baltic Sea has an accentuated phytoplankton phenology, with the summer community flanked by distinct spring bloom from early April to mid-May, and quiescent late autumn—winter populations (Gasiūnaitė *et al*., 2005). To constrain the seasonal effect, we limited the data to the summer season (June–September), which also constrained the interquartile ranges of temperature to 12.2–17.0°C. We only considered samples taken from the upper 5-m layer (station depth permitting), and in rare occasions included pooled samples down to 10 m, but still within the upper mixed layer.

The majority of the phytoplankton samples were counted by only a few persons, and we have interviewed the personnel to assure internally consistent standards regarding sampling, analysis, taxonomic resolution and that there were no major changes in the methodology. After controlling for the major determinants of the community structure, time and seasonality, the personnel change accounted for <4% of the community variation. In the data cleaning phase, we carefully backtracked and harmonized the nomenclature to the latest update in the World Register of Marine Species (Costello *et al*., 2013). All phytoplankton data were species-specific and fully quantitative, with species-specific biovolumes converted to wet weight biomass in μg L^−1^, assuming a density of 1 g mL^−1^.

### Data analysis

#### Community ordination

We first constructed a phytoplankton community data matrix from the biomass values, samples in rows and taxa in columns. The global 4630 × 620 summer community matrix was square root transformed, followed by Wisconsin double standardization (first, species divided by their maxima; second, samples divided by sample totals) and subjected to non-metric multidimensional scaling (NMDS) with two axes (*metaMDS*, *vegan* library in R). The first NMDS axis correlated with the long-term time trend, whereas the second axis correlated with proximity of the shoreline and station depth (a coastal-pelagic gradient). A number of stations were located within the numerous shallow inner bays of the city, which suffered from severe nutrient input during the observed period and were analyzed separately from the more pelagic stations. We used the second NMDS axis to split the dataset into 1115 × 506 inner and 3515 × 547 outer station subsets (10 and 26 stations, respectively), which were analyzed separately thereafter (Fig. 1 and Supplementary Fig. S1, see online supplementary data for a colour version of this figure).

The goal of NMDS is to condense the information from multiple dimensions (e.g. species) into a few, to ease interpretation and visualization. The advantage of NMDS is that it represents the rank ordering relationships amongst samples within a given set of dimensions better than primarily Euclidean distance based eigen-ordinations. The use of rank orders evades hurdles associated with using absolute distance (e.g. sensitivity to transformation), and is thus more flexible and accepts a variety of data types. Because the relationship between data dissimilarity and ordination distance is generally non-linear, NMDS is commonly considered as the most robust unconstrained ordination method in community ecology (Legendre and Legendre, 2012). The site scores from the ordination were further used to assign the stations into three groups, based on k-means clustering (*kmeans*, *stats* library in R).

#### External covariates

The association between the phytoplankton community composition and a set of continuous scale external covariates (Table I) was analyzed with three methods. First, the external covariates were regressed as dependent variables onto the ordination (*envifit*, *vegan* library in R), and visualized as arrows towards the direction of the steepest linear increase of the covariate. The length of the arrow was scaled to be proportional to the explained variance of the regression (*R*^2^).

**Table I:** The associations between the phytoplankton composition and external covariates

	*envfit R* ^2^		*mantel r*		*adonis R* ^2^	
External covariate	Outer archipelago	Inner bays	Outer archipelago	Inner bays	Outer archipelago	Inner bays
Year	0.88	0.9	0.75	0.67	0.18	0.12
Seasonality	0.21	0.003	0.065	0.14	0.009	0.024
P_tot_	0.039	0.36	0.072	0.092	0.081	0.008
N_tot_	0.021	0.2	0.097	0.081	0.04	0.009
Chl a	0.027	0.3	0.11	0.09	0.061	0.008
PO_4_	0.04	0.28	-0.0004	0.091	0.062	0.01
NO_3_	0.002	0.076	0.14	0.023	0.02	0.003
NH_4_	0.03	0.058	-0.007	0.062	0.015	0.005
pH	0.084	0.38	0.250	0.067	0.073	0.011
Salinity	0.062	0.097	0.210	0.079	0.031	0.017
Temperature	0.085	0.013	0.022	0.11	0.006	0.019
Species richness	0.7	0.56	0.46	0.44	0.11	0.092
Species evenness	0.17	0.11	0.17	0.13	0.033	0.022
Phylogenetic diversity	0.091	0.23	0.33	0.14	0.043	0.026

The statistics represent variance explained (*R*^2^) by regressing the covariates onto ordination (*envfit*) or community matrix (*adonis*), or Mantel statistic (correlation coefficient, *r*) between the distance matrices of covariate and the community (*mantel*).

Next, we used two complementary methods, which are independent of the ordination. First, we used Mantel test (*mantel*, *vegan* library in R), which explores the correlation between two distance matrices. The first distance matrix was Bray–Curtis dissimilarity computed on Wisconsin-square root transformed community matrix (the same as used to calculate NMDS above). The second was the Euclidean distance matrix on *z*-scores (mean zero, unit standard deviation) of a given environmental variable. The two matrices were flattened out into vectors and the Pearson correlation coefficient (*r*) was calculated.

A non-parametric analogue of multivariate analysis of variance (MANOVA; *adonis*, *vegan* library in R) was used as a third approach to assess the effect of external variables on the community variation. The method partitions sums of squares using semi-metric and metric distance matrices, and is a robust alternative to ordination methods for describing how variation in community composition is attributed to different external covariates.

The set of external variables included time (measured in years), seasonality (measured as day of the year), physical environmental variables (temperature and salinity) and chemical variables related to eutrophication (total N and P, phosphate, nitrate, ammonium, pH and chlorophyll a concentration; Table I). Furthermore, we used a range of alpha diversity related variables as covariates—species richness, Shannon evenness and phylogenetic diversity. Species richness is a special case of species diversity, which weights common and rare species equally. Jost (2006) generalized species diversity as the “effective number of species,” which is the number of equally-common species to give a particular value of a diversity index. This effective number of species can be calculated as Hill numbers, which results in a profile of diversities along a gradient of *q*—the order of diversity—controlling for the weight given to rare vs dominant species. *q* equal to zero weights all species equally, ignoring the differences in abundance; *q* equal to unity weights species proportionally to their abundance in the community, and *q* > 1 gives more weight to common species. Although overweighting rare species is unusual in ecological literature, we calculated the species diversity profiles for *q* ranging from −0.5 to 2 in order to resolve how rare vs. common species contribute to the phytoplankton community trends (Buckland *et al*., 2017).

Species diversity treats taxa as independent entities—a simplification ignoring the known common evolutionary history of species. To account for this non-independence between species, we calculated species similarity-based diversity profiles for the range of *q* values specified above (*Dqz*, *entropart* library in R). The species similarity matrix was obtained from phylogenetic distance between species (*cophenetic*, *stats* library in R), calculated from a coarse phylogeny based on the taxonomic classification of species in the World Register of Marine Species (Costello *et al*., 2013).

#### Time-series analysis of community composition and alpha diversity

The 52-year trends in phytoplankton community were analyzed with thin-plate regression spline fitted with generalized additive mixed models (*gamm*, *mgcv* library in R). To visualize the decadal-scale trends in phytoplankton community composition, we plotted the scores of the first NMDS axis against observation time for both, inner and outer archipelago stations, superimposed by the *gam* smoother. To visualize the trends in alpha species diversity, we plotted taxon richness and evenness against observation time, superimposed by the respective *gam* smoothers. We used Shannon evenness—the Hill’s ratio, which is the ratio of species diversities of orders *q* = 1 and *q* = 0. Diversity of order 0 is the conventional taxon richness, which ignores the differences in species abundances.

We structured the gam model as:

$$ {y}_i={\beta}_0+{f}_1\left({\mathrm{time}}_{\mathrm{i}}\right)+{f}_2\left({\mathrm{doy}}_{\mathrm{i}}\right)+{\varepsilon}_{\mathrm{i}},\varepsilon =N\left(0,{\delta}^2\varLambda \right) $$

where *y_i_* is the dependent variable—either the observed NMDS first axis score, species richness or evenness of a sample, β_0_ is the intercept, 𝑓_1_ represents the smooth functions of time (measured in years), 𝑓_2_ represents the seasonality from June to September (measured as day of the year) and ε_i_ is the Gaussian error term with zero mean and δ^2^Λ variance (Supplementary Fig. S2, see online supplementary data for a colour version of this figure). Λ is a correlation matrix describing the dependence structure of the residuals. The diagonal elements of Λ are equal to 1, indicating a constant variance of δ^2^ for each residual.

As the response is a time-series, it is plausible that the residuals will not be independent, but include autocorrelation arising from temporal variation in the response, which is not captured by the systematic part of the model. Because the phytoplankton data were irregularly sampled in time we chose the continuous time first-order autoregressive process CAR (1) dependence structure for Λ, which allows for varying temporal distances between residuals when determining the degree of dependence (Elorrieta *et al*., 2019). The CAR(1) describes a process whereby correlation between observations (residuals) declines exponentially with increasing temporal separation.

We might expect the data to have a hierarchical structure, with individual communities nested within blocks represented by the fixed monitoring sampling locations (Fig. 1). Either the intercept, smoother or wiggliness of the smooth term may be subject to grouping. We included the site-specific random effects by using the hierarchical gam (HGAM) extension (Pedersen *et al*., 2019). We started with the simplest model from the HGAM framework (model G in Supplementary Table S1), which included a single common smoother for all observations:

$$ {y}_i={\beta}_0+{f}_1\left({\mathrm{time}}_i\right)+{f}_2\left({\mathrm{doy}}_i\right)+\zeta \mathrm{stn}+{\varepsilon}_{\mathrm{i}},\kern0.33em \varepsilon =N\left(0,\kern0.33em {\delta}^2\varLambda \right) $$

where ζstn is the random effect for sampling station. The temporal evolution of communities could take different trajectories in various stations (model GS in Supplementary Table S1). To allow for different smoothers per station we added complexity to the previous model:

$$\begin{array}{lc} {y}_i&={\beta}_0+{f}_1\left({\mathrm{time}}_i\right)+{f}_2\left({\mathrm{doy}}_i\right)+{f}_{\mathrm{stn}}\left({\mathrm{time}}_i\right)+{\varepsilon}_{\mathrm{i}}, \\ &\varepsilon =N\left(0,\kern0.33em {\delta}^2\varLambda \right) \end{array}$$

where 𝑓_stn_(time*_i_*) is the smoother for the given station. Although this model allows for different shapes of the station specific smoothers, it does penalize the differences from the common smoother. Finally, we could assume different rate of temporal change in the various stations (model GI in Supplementary Table S1). In the HGAM framework this would translate into a single common smoother plus group-level smoothers with different wiggliness.

We fitted the models separately for the inner bay stations, and for the outer archipelago region. Mathematically the models could be extended not to include a common smooth term, but we considered these solutions ecologically unlikely. Given the geographical proximity of sampling stations, it is plausible to assume a shared common pool of information.

#### Significance of the trendlines

The fitted models represent the variation of the mean of the response variable in time. We were specifically interested in the long-term trend of phytoplankton community over years, so we focused on the smooth 𝑓_1_. For a simpler interpretation of the fitted trends, we identified periods along the trendline where the slope, i.e. the rate of change in the trend, was significantly different from 0, either increasing or decreasing. For this we computed the first derivatives of 𝑓_1_ using the method of finite differences as in Curtis and Simpson (2014). In short, fitted values of the trend were obtained from each model for a grid of 200 equally spaced time points over the period of observation. This grid was shifted in time by a minute amount and fitted values of the trend again determined from the model. The differences between the two sets of fitted values, divided by the difference in time, yield the first derivatives of the trend. Standard errors for the first derivatives were also computed and a 95% point-wise confidence interval on the derivative determined. Whenever the confidence intervals of the slope did not include zero, the slope was considered significant at 95% level, either increasing or decreasing.

#### Trends in beta diversity

There are numerous dissimilarity coefficients suitable for beta diversity assessment (Tuomisto, 2010; Legendre and De Cáceres, 2013). We adopted the Sørensen based dissimilarity index due to the virtue that it can be decomposed into two fractions, one derived from differences between species richness (species gain and loss) and the other from differences due to species replacement (also known as species turnover; Legendre, 2014; Podani and Schmera, 2016). Replacement refers to the phenomenon that species tend to replace each other along ecological gradients. Richness difference refers to one community including a larger or smaller number of species than another. The quantitative form of the Sørensen index, also termed as percentage difference, is mathematically equivalent to the Bray–Curtis dissimilarity:

$$ \mathrm{Beta}=\left(B+C\right)/\left(2A+B+C\right)=\mathrm{Replacement}+ AbDif $$

where *A* designates the sum of the minimum abundances of the various species, each minimum being the abundance at the site where the species is the rarest. *B* is the sum of abundances at site 1 minus *A*, and *C* is the sum of abundances at site 2 minus *A* (Legendre, 2014).

The total beta diversity between two communities was decomposed into replacement (turnover) and richness difference, termed as abundance difference in its quantitative form:

$$ {\displaystyle \begin{array}{l}\mathrm{Replacement}=2\times \min \left(B,C\right)/\left(2A+B+C\right)\\{}\mathrm{Abundance}\ \mathrm{difference}=\left|B-C\right|\left(2A+B+C\right)\end{array}} $$

We calculated the beta diversity, as well as the replacement and richness difference components between all pairwise combinations of the 4630 square root transformed communities (i.e. 4630 × (4630–1)/2 = 10 716 135 indices; *beta.div.comp*, *adespatial* library in R, with quant = TRUE and coef = “S” options), and used aggregated arithmetic means to represent the specific time trends.

#### Distance decay

Distance decay is used to characterize the frequently assumed exponential decay in ecological similarity of communities between two sites as a function of their distance apart along a spatial or temporal gradient (Soininen *et al*., 2007; Soininen, 2010). The exponential distance–decay curve has the following formula:

$$ s={\alpha \mathrm{e}}^{-\beta \mathrm{d}} $$

where α and β are parameters to be estimated, *d* is the difference between the sampling time in years, and *s* is the compositional similarity between two sites, expressed here as 1—Bray–Curtis dissimilarity. We fitted the distance–decay curve using a generalized linear model (*glm*) with binomial observation error and a log link function (Millar *et al*., 2011). From the model fit we calculated similarity at zero time difference as exp(α), and halving time as −log(2)/β. The halving time reflects the rate of species turnover per unit time, being thus a measure of the time scale-dependency of the beta diversity.

## RESULTS

**Fig. 2:**
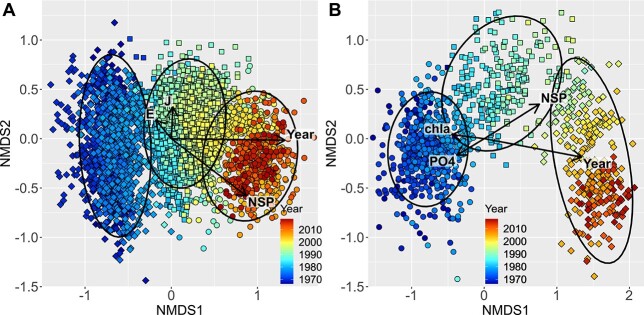
NMDS ordination of phytoplankton communities. The arrows show the direction and strength (proportional to the length of the arrow; see Table I) of external gradients superimposed on the ordination: sampling year (Year), season—measured as day of the year (J), sample species richness (NSP), evenness (E) and two eutrophication related environmental variables—chlorophyll a (chla) and phosphate (PO_4_). The symbols are color-scaled with sampling year and the symbol shape corresponds to one of the k-means three cluster group. Ellipses surround 95% of the cluster groups. The panels refer to the pelagic sites (**A**; 3515 samples × 547 species; stress = 0.2) and inner bay stations (**B**; 1115 samples × 506 species; stress = 0.17).

The NMDS ordination of the phytoplankton communities revealed all but random distribution of communities in time (Fig. 2). Notably, in the pelagic stations, as well as in inner bays, the first ordination axis was close to perfectly aligned with sampling year, and had a strong relationship with species richness. Yet there were also substantial differences between the two sub-areas, as eutrophication-related variables (total and dissolved nutrients, chlorophyll a concentration, pH) were strongly associated with the community composition in the inner bays, but not so in the more pelagic stations (Table I). Not surprisingly salinity and temperature, which are commonly strong predictors of phytoplankton species composition, had only a meagre association with the community ordination, probably due to the limited variation of these variables, as the geographical and seasonal scope of the study was constrained. Though limited to summer months, season still had the third strongest association with the ordination in the outer archipelago stations, next to long-term temporal effect and species richness.

Species richness was the alpha diversity metrics with the strongest relation to the community structure (Table I). The strongest association was with the diversity of order 0, which ignores the differences in species abundances (Supplementary Fig. S3, see online supplementary data for a colour version of this figure). In the inner bays the strongest associations were with order of diversity slightly above 0. Species diversity had a stronger relationship with community structure than phylogenetic diversity. Phylogenetic diversity was relatively more influential in the inner bays compared with the outer archipelago.

**Fig. 3:**
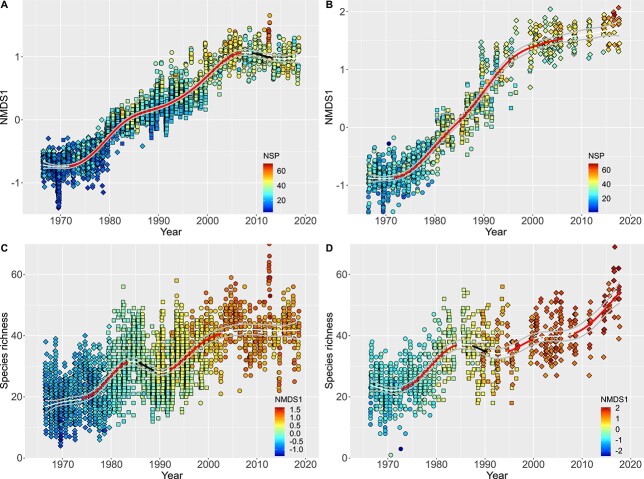
Time trends of the scores of the first ordination axis (**A**, **B**) and phytoplankton species richness (**C**, **D**). In A and B the smooth lines shows the NMDS1 GAM fit of the year term: *F* = 1503, edf = 8.5, *n* = 3515, *R*^2^ = 0.91; *F* = 1331, edf = 7.5, *n* = 1115, *R*^2^ = 0.94 for outer archipelago (A) and inner bays (B), respectively. In (C) and (D) the smooth lines show the GAM fit of species richness trends: *F* = 178, edf = 8.2, *n* = 3515, *R*^2^ = 0.56; *F* = 140, edf = 7.8, *n* = 1115, *R*^2^ = 0.61 outer archipelago (C) and inner bays (D), respectively. The gray lines show the standard errors of the smooth. The red sections of the smooth lines highlight significant (*P* < 0.05) positive slopes, the black sections significant negative slopes, and white stripes non-significant slope. Symbols shape corresponds to the three k-means clusters (as in Fig. 2). Color-codes represent by sample species richness (A and B) or NMDS1 scores (C and D).

Plotting the first ordination score against time revealed the long-term evolution of the phytoplankton community (Fig. 3A and B). Notably, this evolution changed course in time. The long unidirectional drift between 1970s and 2000s, with a statistically significant slope at *P* = 0.05 level, was flanked with different trajectories in the 1960s and in the outer archipelago conspicuously also during the last 1.5 decades (Fig. 3A). The trend in the inner bays revealed decreasing rate of change towards the end of the time-series (Fig. 3B).

We modeled the first ordination axis scores as a function of time with additive mixed models (*gamm*, *mgcv* library in R) with CAR(1) correlation structure, conditional to inner and outer stations. Models without an autocorrelation structure always performed significantly worse (*P* < 0.0001; Supplementary Table S1). Akaike’s Information Criterion (AIC) also favored the CAR(1) models (Supplementary Table S1). Furthermore, comparing the additive models revealed significant hierarchical structure in the phytoplankton data, based on the fixed sampling locations (Supplementary Table S1 and Supplementary Figs S4 and S5, see online supplementary data for a colour version of figures). There was no universally best model for all the independent variables and therefore the smooths presented in Fig. 3 do not account for random effect variation caused by discrete stations, and are based on model-C, which represents the most important and easy to interpret variation.

Ordination scores, although sensitive to trends in time, are not informative of the specific type of change in the community composition. The strong relationship between species richness and the ordination suggest that changes in alpha diversity could be instrumental. Indeed, analyzing species richness as a function of time revealed a complex, non-monotonic pattern (Fig. 3C and D). The overall trend was increasing species richness, with notable and significant reversal period from mid-1980s to early 1990s. In the outer archipelago the increase in species richness conspicuously leveled off since early 2000s (Fig. 3C), which agrees with the temporally more stable overall community composition during the same period (Fig. 3A). However, in the inner bays, the species richness continued to increase (Fig. 3D), which is in line with the continued shift in the overall community composition (Fig. 3B).

Species evenness, assessed here as the Shannon evenness (Hill’s ratio), varied with significant directional changes, but had no universal trend (Supplementary Fig. S6, see online supplementary data for a colour version of this figure). Evenness tended to decrease in the outer archipelago and increase in the inner bays, but the variance explained (*R*^2^) was low (Supplementary Fig. S6, see online supplementary data for a colour version of this figure). In the outer archipelago, evenness was negatively correlated with species richness (Pearson *r* = −0.42, *P* < 0.001, *df* = 3513), whereas there was a weak positive correlation in the inner bays (Pearson *r* = 0.15, *P* < 0.001, *df* = 1113). Other species evenness metrics (Pielou evenness and Simpson’s evenness) gave overall similar results, but had more variable residual distribution (data not shown).

**Fig. 4:**
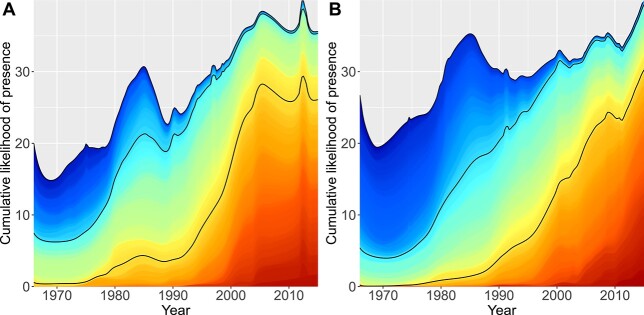
Cumulative likelihood of species presence along time gradient in outer archipelago (**A**) and inner bays (**B**). The cumulative likelihood of each species is plotted in the order of their respective temporal occurrence optima, shown with the color code. Cold hues represent species with highest occurrence likelihood in early period, and warm hues represent species with later occurrence optima. The overall increase represents the increase in species richness, which translates into overall higher cumulative likelihood of presence. The black curves denote boundaries between the three groups of species as in Supplementary Tables S2 and S3.

Although excluding rare species (occurrence in < 5 samples), we modeled the likelihood of species presence along a grid of regular time points using GAM with binomial error distribution. We then ordered the species chronologically according to their preferred occurrence time (calculated as center of gravity along the regular time gradient), and plotted the cumulative likelihood of presence to visualize the temporal turnover of species (Fig. 4). Next, we splitted the species into three equal sized groups, based on their preferred occurrence time, and tabulated the number of species in major algal classes within each group. Thus species falling into the first group tend to have their occurrence biased towards early years, species in the third group are relative newcomers, and species in the second group have high occurrence likelihood in the middle of the period or occurred relatively evenly throughout the 52 years. Diatoms and chlorophytes revealed a monotonically decreasing trend of species richness along the three temporal groups, whereas Trebouxiophyceae and dinoflagellates had a monotonically increasing trend (Supplementary Table S2). Also cyanobacteria had a monotonically increasing trend in the outer archipelago zone, whereas in the inner bays the variation was not monotonical. Supplementary Table S3 lists three most frequent species within each group and major phytoplankton class.

### Beta diversity

**Fig. 5:**
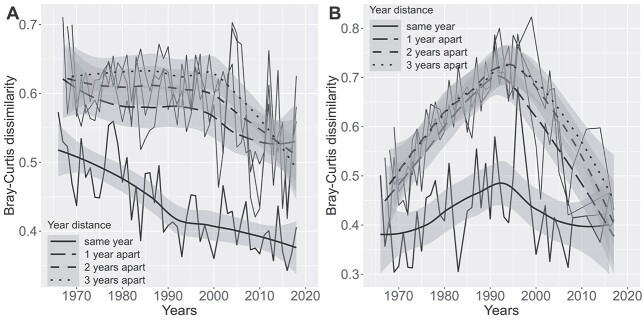
Temporal trends in phytoplankton beta diversity in the outer stations (**A**) and inner bays (**B**). The thin lines connect points of annual means, superimposed by thick smooth line with shaded area representing standard errors. The annual means were calculated from pairwise Bray–Curtis dissimilarity, constrained to sample pairs from within the same month and sampling location to control for seasonal and spatial variation. The solid smooth line was further constrained to samples from the same year; the dashed smooth lines were constrained to have 1, 2 or 3-year difference.

We calculated the mean pairwise beta diversity, measured as Bray–Curtis dissimilarity, for each year, constraining the sample pairing to within the same year, month and sampling location. A decreasing trend was revealed while controlling for the seasonal (month) and spatial (sampling location) variability, with a potential breakpoint in early 1990s, in the pelagic locations (Fig. 5A). At the inner bay stations we found a hump-shaped pattern with a peak in early 1990s (Fig. 5B).

Next, we repeated the averaging by forcing a 1, 2 and 3-year difference between the samples, while still controlling for seasonal and spatial variation as above. Forcing a 1 year difference between the samples increased abruptly the beta diversity, whereas subsequent years increased the diversity with smaller increments (Fig. 5). In our context it is the difference between the intra-annual and interannual components, which inform about the pace of temporal community change on a longer time scale.

**Fig. 6:**
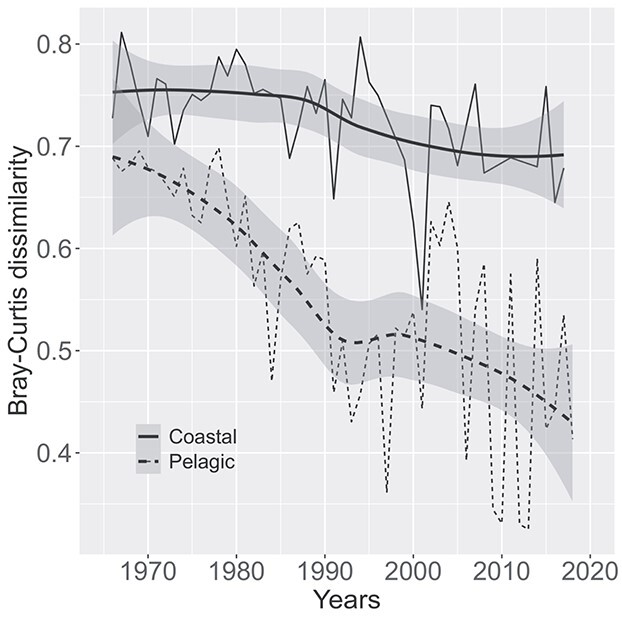
Inter-station temporal trends in phytoplankton beta diversity in the outer stations and inner bays. The annual means of Bray–Curtis dissimilarity, connected with thin lines and superimposed by thick smooth lines with gray shaded standard errors, were constrained to single year and month, but different sampling stations. There is a notable decreasing spatial beta diversity trend in the pelagic location, indicating homogenization of communities, whereas the communities in the inner bays remain idiosyncratic.

We could ask if the phytoplankton community assembly was governed by local processes or by broader general trends. To answer this, we calculated pairwise beta diversity by constraining the sample pairs to be within the same year and month, but from different sampling locations. Consistently high between-station beta diversity in the inner bays suggests the predominance of local community assembly processes (Fig. 6). In contrast, the inter-station beta diversity in the outer archipelago was lower and had a visible decreasing long-term trend, indicative of significant homogenization of the phytoplankton community (Fig. 6).

### Beta diversity decomposition

The mean pairwise beta diversity across the whole investigated period, constrained within the same year, month and station, was 0.46 and 0.39 in the outer archipelago and inner bays, respectively (Fig. 5). The mean species turnover components were 0.30 and 0.25, respectively, and the richness difference components 0.16 and 0.14. Thus in both regions, species turnover dominated the beta diversity, being 64–65% of the total beta, leaving 35–36% to richness differences.

**Fig. 7:**
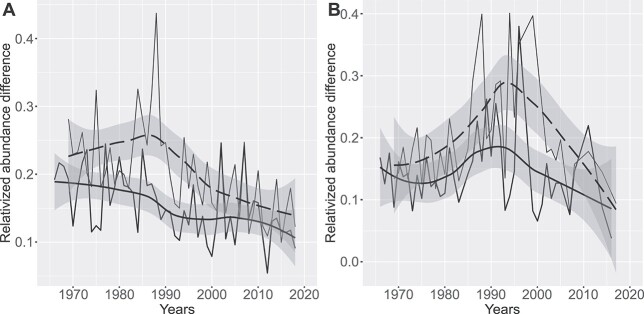
Time trend in the richness component of the beta diversity, estimated as relativised abundance difference, in the pelagic stations (**A**) and inner bays (**B**). Thin lines link annual means, constrained by sampling location and month. Solid smooth line is constrained to sample pairs of the same year, dashed line to samples with 3-year difference.

The trend of the decomposed richness difference revealed an interesting pattern. Constrained by season and location, the intra-annual richness difference had a slight decreasing trend in the outer archipelago, while remaining steady in the inner bays (Fig. 7). The inter-annual richness difference (3-year difference) had a maximum in the middle of the period, significantly higher than the intra-annual background (Fig. 7). These humps correspond to periods with excessive species richness differences in a 3-year time scale.

### Distance decay

**Fig. 8:**
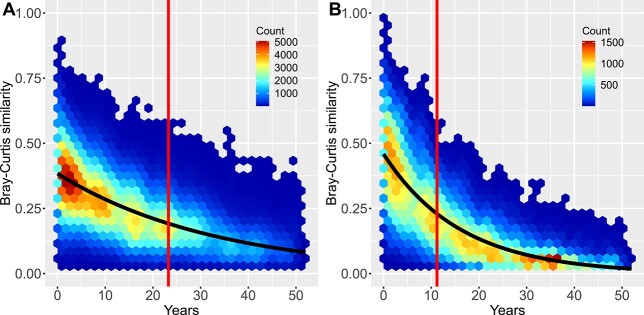
Distance decay of the compositional similarity between phytoplankton summer communities in the pelagic stations (**A**) and inner bays (**B**). The pairwise Bray–Curtis similarity, calculated as 1-dissimilarity, were constrained to same sampling location, but separated in time. The hexagonal heatmap cells contains counts (*n* = 531 848 and 162 170 for outer archipelago and inner bays, respectively). The black solid line is a generalized linear model (*glm*) fit to the data with binomial observation error and a log link function. The vertical red lines show the halving time—the time interval when Bray–Curtis similarity at the intercept (time zero) had decreased to half the value.

The initial similarity at time 0, calculated as the intercept in the *glm* model, was 0.38 in the outer archipelago and 0.46 in the inner bays (Fig. 8). Across both subsets, compositional similarity showed a pronounced decay in time, but the rate of decay (quantified as halving time) differed. The similarity of phytoplankton species assemblages declined by 50% every 23.5 years (95% CI: 23.2–24 years) in the outer archipelago (Fig. 8A), but much more rapidly, within 11.3 years (95% CI: 11.1–11.5 years) in the inner bays (Fig. 8B).

## DISCUSSION

Over the half-century, the coastal phytoplankton community has been highly dynamic in the Helsinki archipelago, the Baltic Sea. The pronounced shifts and reversals of community composition and species richness temporal trajectories since the late 1960s have remarkably leveled off since the turn of the century—a change, which was not discernible from our earlier studies with a decade shorter dataset (Olli *et al*., 2011, 2014). Microscopy-based phytoplankton monitoring data have inherent variability (Thackeray *et al*., 2013), also evidenced by the high mean beta diversity between-samples taken at close proximity in time and space. Despite the local variability, the wealth and quality of the data enabled to reveal conspicuous long-term time trends in community composition, and several facets of alpha and beta diversity. The causes of the observed phytoplankton trends are not definite. In the following, we discuss human aided dispersal of species by the ever increasing shipping into an unsaturated brackish species pool of the geologically young Baltic Sea, and eutrophication history of the region as plausible drivers.

### Trend of increasing species richness

The long-term increase in species richness may appear unusual in today’s era of global human dominance and influence, in which changes in biodiversity are commonly reported to be large and negative (McGill *et al*., 2015; He and Silliman, 2019). However, despite global decline, there is some evidence that biodiversity at regional or local scales can be steady or even increasing (Sax and Gaines, 2003; Pilotto *et al*., 2020). In particular, marine coastal ecosystems have experienced increasing trends in local species richness across groups and systems globally (Elahi *et al*., 2015), which contrasts the predominantly steady local-scale time trend in terrestrial systems according to several meta-studies (Vellend *et al*., 2013; Dornelas *et al*., 2014).

The conspicuous long-term increase in phytoplankton species richness demonstrated here is in line with an earlier study at the scale of the whole Baltic Sea (Olli *et al*., 2014). We fully acknowledge that species richness is sensitive to changes in counting effort and taxonomic skills of the microscopist. This potential bias is difficult to fully eliminate, but can be alleviated by scaling species richness with resource (nutrient) use efficiency RUE, as in Ptacnik *et al*. (Ptacnik *et al*., 2008) and Olli *et al*. (Olli *et al*., 2014).

Increase in local richness occurs when species immigration exceeds extinctions. This may happen when human-mediated dispersal of alien organisms is high (e.g. through ballast water transport; Wonham and Carlton, 2005), or global changes allow species to invade areas in which they previously could not persist (Elahi *et al*., 2015). The Baltic Sea is one of the most heavily trafficked seas in the world; ~15% of the world’s cargo transportation takes place in the area with an increasing trend over the decades (Stankiewicz & Vlasov, 2009). Further, the Baltic Sea is geologically young—ca 7500 years as a brackish basin and ca 2000 years at the present salinity level (The Baltic Sea: History and geography, 2009). The Baltic Sea species pool is undersaturated, leading to frequent invasions from the global brackish meta-populations (Olenina *et al*., 2010).

We identified species richness to be the most influential alpha diversity metric describing the community change. Species or phylogenetic diversity metrics, which accounted for differences in the species abundances (i.e. order of diversity *q* ≠ 0), had considerably weaker association with the phytoplankton community change than species richness (Supplementary Fig. S3, see online supplementary data for a colour version of this figure). Evenness indices showed a weak pattern in mean values over the 52 years. This suggests that algal bloom frequency, which should be reflected in the evenness, had not changed systematically over time.

### Trends in beta diversity and eutrophication

Species richness has been criticized as a poor metric of biodiversity trend, as it does not account for compositional turnover (Koricheva *et al*., 2017; Hillebrand *et al*., 2018). With little or no change in richness, immigrations and extinctions can be equally intense, leading to outright turnover of species composition—the other major component of beta diversity besides richness change (Wonham and Carlton, 2005). In our time-series, the mean species richness change accounted for ca 35–36% of the beta diversity, which is significant, but indicates that overall the biodiversity change of the phytoplankton community was dominated by species turnover. High species turnover indicates that immigration of new species and regional extinctions are important processes in maintaining the local phytoplankton diversity (64–65%).

Globally, the long-term trends in temporal and spatial beta diversity are poorly studied across biomes and groups (McGill *et al*., 2015). Here, we show that the mean inter-annual temporal beta diversity was considerably higher than the intra-annual background trend (Fig. 5). In the outer archipelago, the temporal beta diversity trend was remarkably steady until mid-1990s—a time point when the difference from the local intra-annual background beta diversity was the largest. This indicates that the temporal change in phytoplankton biodiversity accelerated up to a turning point in mid-1990s, followed by decreasing pace thereafter. The turning point in mid-1990s was even better discernible in the mean temporal beta diversity trend in the inner bays (Fig. 5B), forming a hump-shaped trajectory in the background of a more steady intra-annual beta diversity trend. Concomitantly, the regional differences in eutrophication related variable correlation with phytoplankton community ordination (Fig. 2 and Table I) likely stemmed from the starker hyper-eutrophication history in the inner bays (Finni *et al*., 2001; Vaalgamaa, 2004).

We propose that the trends in temporal beta diversity were in excess to what could be explained by a neutral drift alone, and that community turnover was likely directional due to anthropogenic factors like eutrophication. The Baltic Sea, and the Gulf of Finland in particular, have suffered from severe eutrophication pressure during the last century (Vahtera *et al*., 2007; Andersen *et al*., 2017; Heiskanen *et al*., 2019). The eutrophication pressure in the northern Baltic Sea peaked in the mid-1980s, followed by a gradual and slow recovery in response to stringent management measures imposed by the surrounding countries, the Helsinki metropolitan area included. We suggest that that the trend in temporal beta diversity reflects the adaptation to the changing environment.

The eutrophication pressure was particularly sharply accentuated in the shallow inner bays of the Helsinki City, which were very eutrophied already in the beginning of the 20th century (Finni *et al*., 2001; Vaalgamaa, 2004). In the 1950s, the city population had increased to 370 000 inhabitants, and a pronounced increase in nutrient accumulation occurred. The peak years in the loading and trophic status could have been already in 1960s, prior to the regular monitoring programs (Vaalgamaa, 2004). Water column nutrient concentrations peaked in the 1970s, when 11 separate wastewater treatment plants released treated sewage water to inland bays around the city, adding to the already intense internal loading.

Up until 1987 waste waters in the region were treated in several smaller treatment plants and effluents were discharged into the shallow coastal zone in the inner bays. From late 1987 onwards until 1994, Helsinki progressively switched to a centralized and more efficient wastewater treatment system and the purified wastewaters were diverted to the outer archipelago through a tunnel with an opening at the sea floor at 20-m depth (Vaalgamaa, 2004). Subsequently, in the following years the water quality in the inner bays improved significantly (Supplementary Fig. S7, see online supplementary data for a colour version of this figure). This late 1980s to mid-1990s turning point was well reflected in the temporal beta diversity trend of the phytoplankton community (Fig. 5B). Before mid-1990s the temporal beta diversity increased rapidly, indicating accelerating inter-annual pace of phytoplankton community change. After the diversion of treated wastewater to the outer archipelago, the change in the phytoplankton community composition gradually slowed down, to the point in recent years where it does not differ from the background intra-annual beta diversity. The trajectory of eutrophication was thus well reflected in the long-term phytoplankton biodiversity trend.

### Phytoplankton community homogenization

There is growing evidence of spatial taxonomic homogenization from local to global scales (McKinney and Lockwood, 1999; Baiser *et al*., 2012; Li *et al*., 2020)—a process where the community composition at different sites becomes increasingly similar to each other in time. We found that the mean beta diversity between the outer archipelago stations, i.e. spatial beta diversity, decreased significantly throughout the investigation period (Fig. 6). This indicates substantial homogenization of the phytoplankton community composition. Homogenization, however, was not the case in the inner bay station as the spatial beta diversity remained steadily high. This suggests that the community assembly in the inner bays was consistently governed by local processes, with restricted dispersal between the meta-communities.

Spatial homogenization is often seen as a human-driven process, like species transport in ballast water associated with shipping traffic (Drake and Lodge, 2004), but also the range expansion of generalist species associated with warmer temperatures (Menéndez *et al*., 2006). Further climate change may lead to further homogenization of ecological communities, which has recently led to the proposition of yet another new era—*homogenocene*—the epoch of biological re-shuffling in ecosystems (Padial *et al*., 2020).

Decreasing spatial beta diversity is also linked to regional increase in species richness—immigration is frequently dominated by the spread of ubiquitous generalist species (Finderup Nielsen *et al*., 2019). Increasing species richness due to the immigration of common and widespread generalist species leads to spatial homogenization of the community composition. Immigrating species, even if few in number, spread often widely, and their contribution to local species pool (alpha diversity) may be offset by their homogenizing impact on composition and beta diversity (Kortz and Magurran, 2019). Furthermore, if species turnover leads to replacement of the regionally unique specialist species by the common generalists, homogenization of species composition between regions may lead to impoverishment of the regional scale biota. This has led the discussion from simply the quantity of biodiversity to also include the quality of biodiversity (McGill *et al*., 2015). Our results support earlier assertion that changes in species richness alone are not sufficient to detect biodiversity response to external perturbation when compositional change is in excess to predictions of ecological theory (Hillebrand *et al*., 2018; Kortz and Magurran, 2019).

### Distance decay

In a cross-system and -group meta-analysis Dornelas *et al*. (Dornelas *et al*., 2014) found that globally ca 10% of the species are replaced by new species every year—a rate well in excess of the expectation of null models of assemblage change. The distance-decay approach adopted here revealed a rapid temporal compositional turnover, well in excess of the global average estimated by Dornelas *et al*. (Dornelas *et al*., 2014). The effect size of initial Bray–Curtis similarity at zero time distance, 0.39 and 0.45 in the outer archipelago and inner bays, respectively, was low compared with 0.88 estimated by a global meta-analysis across systems and groups (Soininen *et al*., 2007), but agreed well with planktonic diatom communities [(0.38); Wetzel *et al*., 2012]. Small organisms with their short generation time respond more rapidly to fine scale variation in the environment, and therefore will have lower similarity at small distances.

Distance decay reflects both neutral drift and species sorting due to changing environmental conditions. Dornelas *et al.* (Dornelas *et al*., 2014) found that neutral drift was two orders of magnitude smaller than the temporal turnover in species composition found in empirical, real-world datasets. It was thus likely that the temporal turnover of phytoplankton community was overwhelmingly driven by environmental change. The twice shorter halving time of the community composition in the inner bays compared to the outer archipelago reflects the more accentuated environmental changes, particularly the adaptation to and recovery from stark eutrophication pressure.

## CONCLUSIONS

Based on over half a century monitoring time-series we uncovered high temporal dynamics of phytoplankton community structure in a confined coastal area—the Helsinki archipelago, the Baltic Sea. For almost four decades the phytoplankton community and species richness have not been in a steady state, but rather in a continual change, which has conspicuously stabilized since the early 2000s.

Variation in species richness, with a significant net increase, was the single most influential alpha diversity measure associated with the trend in community composition. Yet the variation in species richness accounted for no more than 35–36% of the mean between-sample beta diversity; the rest being explained by species turnover.

Trends in spatial beta diversity revealed discernible homogenization of the communities in the outer archipelago zone, which is concurrent with the global trends in local and regional biodiversity patterns. The communities in the inner bays retained high spatial beta diversity throughout the half century time interval, suggesting high idiosyncrasy and dispersal limitation.

The overall community turnover in the inner bays—11.3 years halving distance of community similarity—was more accentuated than in the outer archipelago with 23.6 years halving distance. This reflects the adaptation of phytoplankton community to the more severe eutrophication pressure and environmental change in the shallow coastal bays.

Our study emphasizes the importance of compositional turnover and beta diversity in complement to species richness and alpha diversity metrics when assessing patterns of biodiversity change.

## Supplementary Material

Olli_Trends_Summer_Helsinki_SupplR2_fbac029

## Data Availability

The data and R code for this study is available at: https://github.com/kalleolli/HelSummerTrends
