# Supplementary material for: Half-century trends in alpha and beta diversity of phytoplankton summer communities in the Helsinki Archipelago, the Baltic Sea
Source: J Plankton Res. 2022 Jun 12;45(1):146–62. doi: 10.1093/plankt/fbac029 (PMC9897023; doi:10.1093/plankt/fbac029)
Supplement: Olli_Trends_Summer_Helsinki_SupplR2_fbac029 [file olli_trends_summer_helsinki_supplr2_fbac029.pdf]

Half-century trends in alpha and beta diversity of phytoplankton summer communities in the Helsinki Archipelago, the Baltic Sea

Kalle Olli

Emil Nyman

Timo Tamminen

Supplementary material

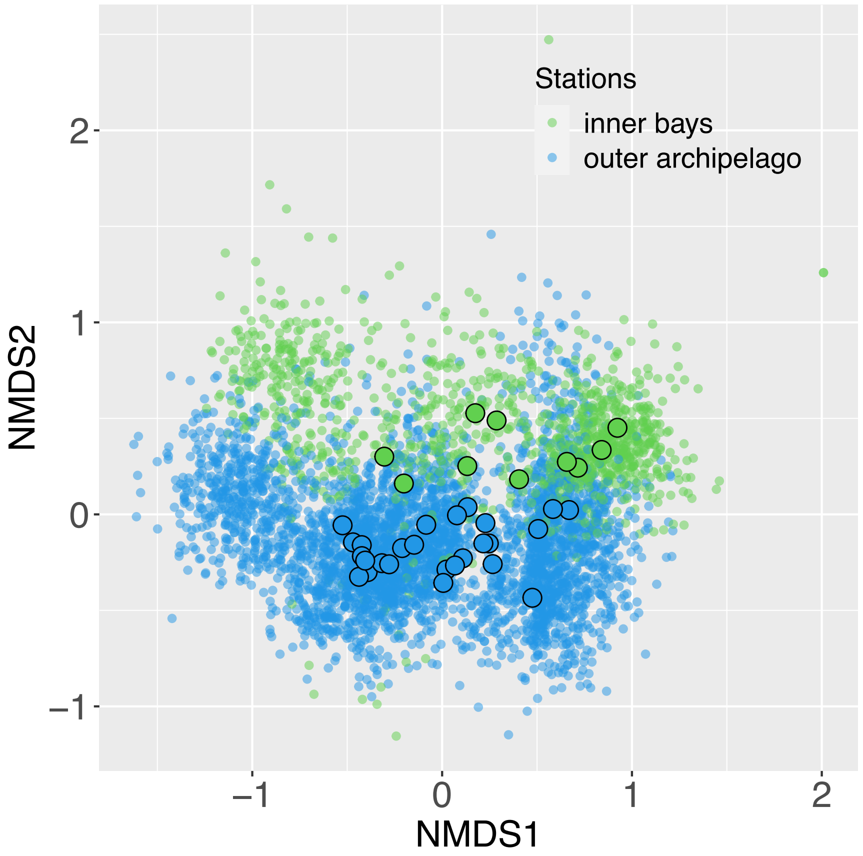

Figure S1. Joint NMDS ordination of all phytoplankton communities (4630 samples × 619 species) from the Helsinki archipelago (1966 2018). NMDS2 correlates with the coastal-pelagic gradient. Large symbols denote the centres of gravity of each station, which were used to split the 36 stations into 10 inner bay and 26 outer archipelago ones according to their NMDS2 scores.

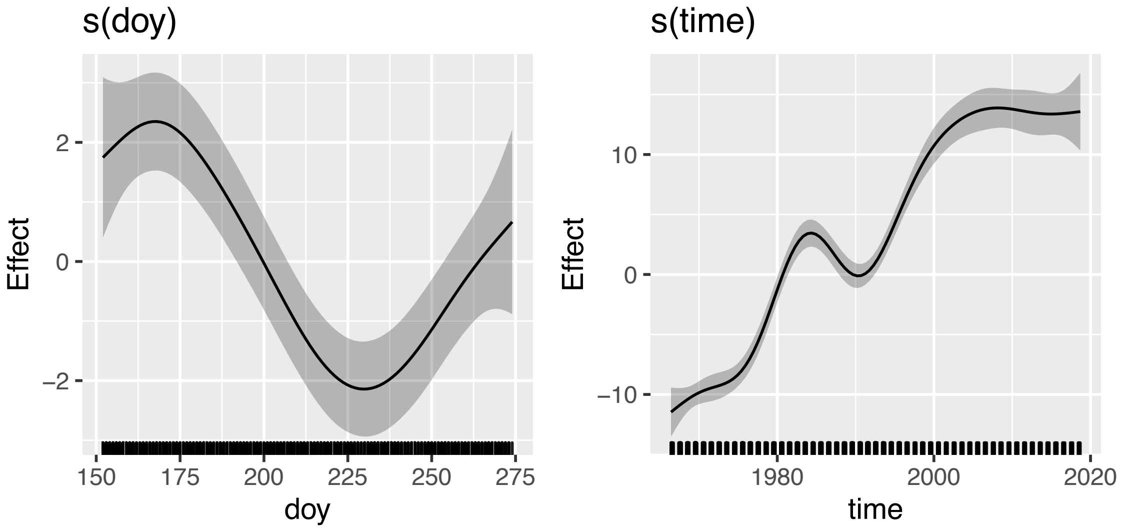

Figure S2. The fitted additive model, visualising the effects of season, measured as the day of year ($f_2(\text{doy}_i)$) and long term trend ($f_1(\text{time}_i)$) for the species richness in the outer archipelago phytoplankton time series. The smooths are centred around 0 to allow them to be identified separately from the model intercept. The shaded area represents the 95% confidence intervals on the fitted smoothers, depicting the uncertainty in the estimate of the smooth term. Note that the season is restricted to summer months – June to September.

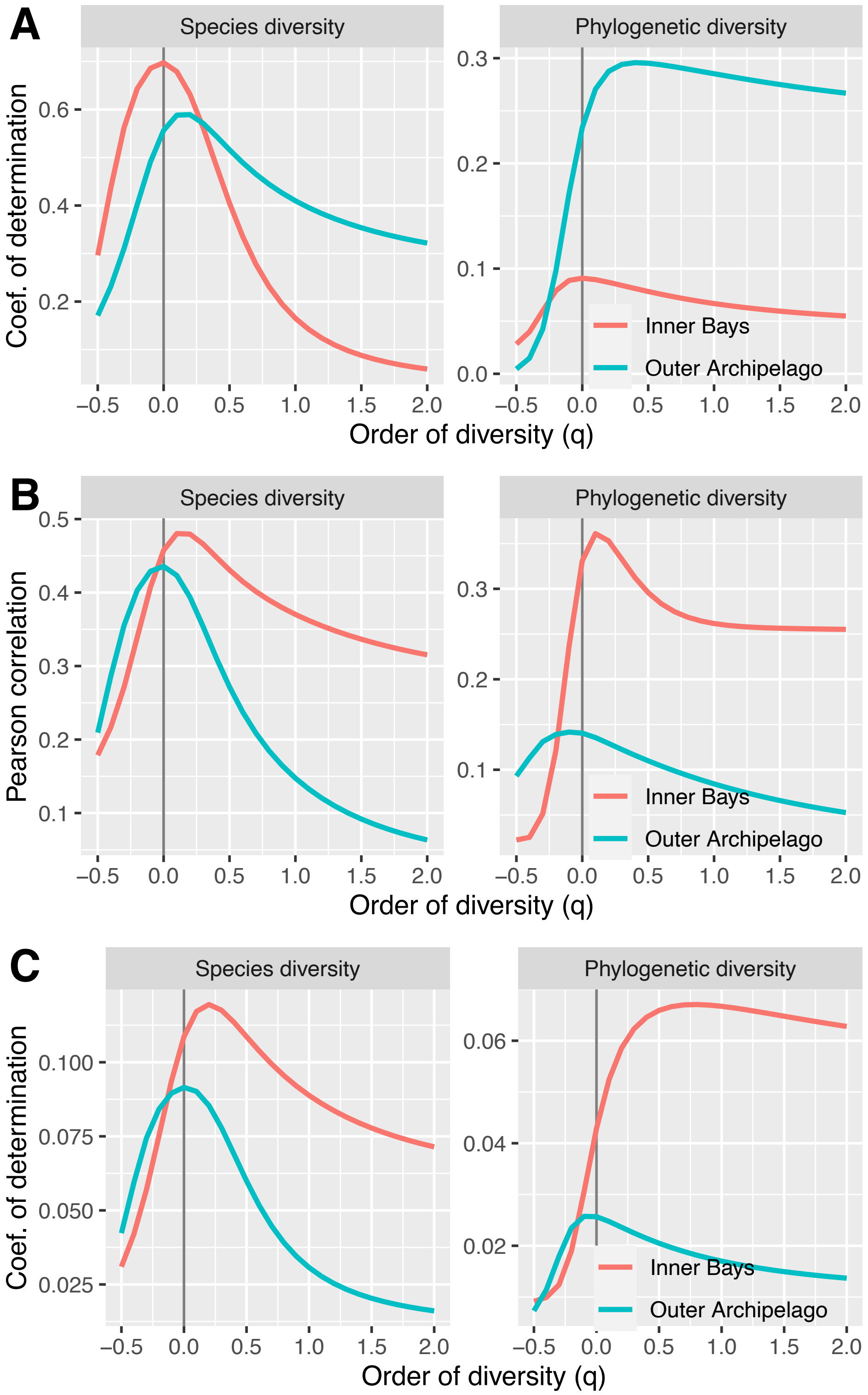

Figure S3. Associations between phytoplankton diversity profiles and community composition in the outer archipelago and inner bays. A – *envfit* linear regression coefficients of determination between diversities and the NMDS ordination scores. B – *mantel* correlation coefficients between Bray-Curtis community dissimilarity and distance matrices of diversities. C – *adonis* regression coefficients of determination between species diversities and community structure. The vertical line represents the diversity of order 0, which treats common and rare species equally.

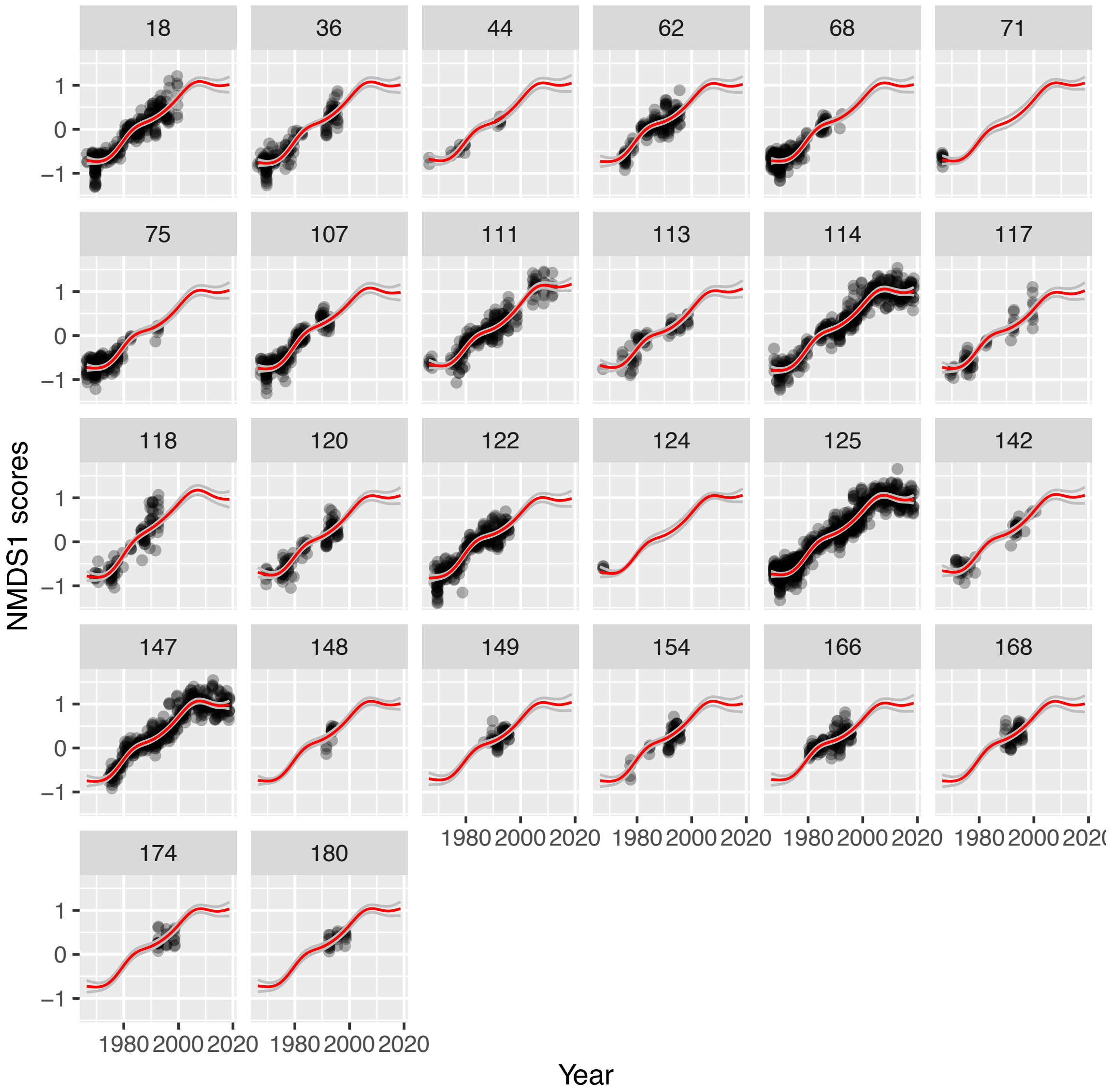

Figure S4. Hierarchical additive model fit of NMDS1 scores against time in outer archipelago stations (panels). The model has a common intercept and smooth term, but allows variation in the intercept and shape of smooth for each station (model GS). Station specific departures from the common intercept and smooth term are penalized. The red line represents the model fit of the long term trend; grey lines represent two times standard error of the fit. Original data points are plotted in the background with transparency. The station labels follow Figure 1.

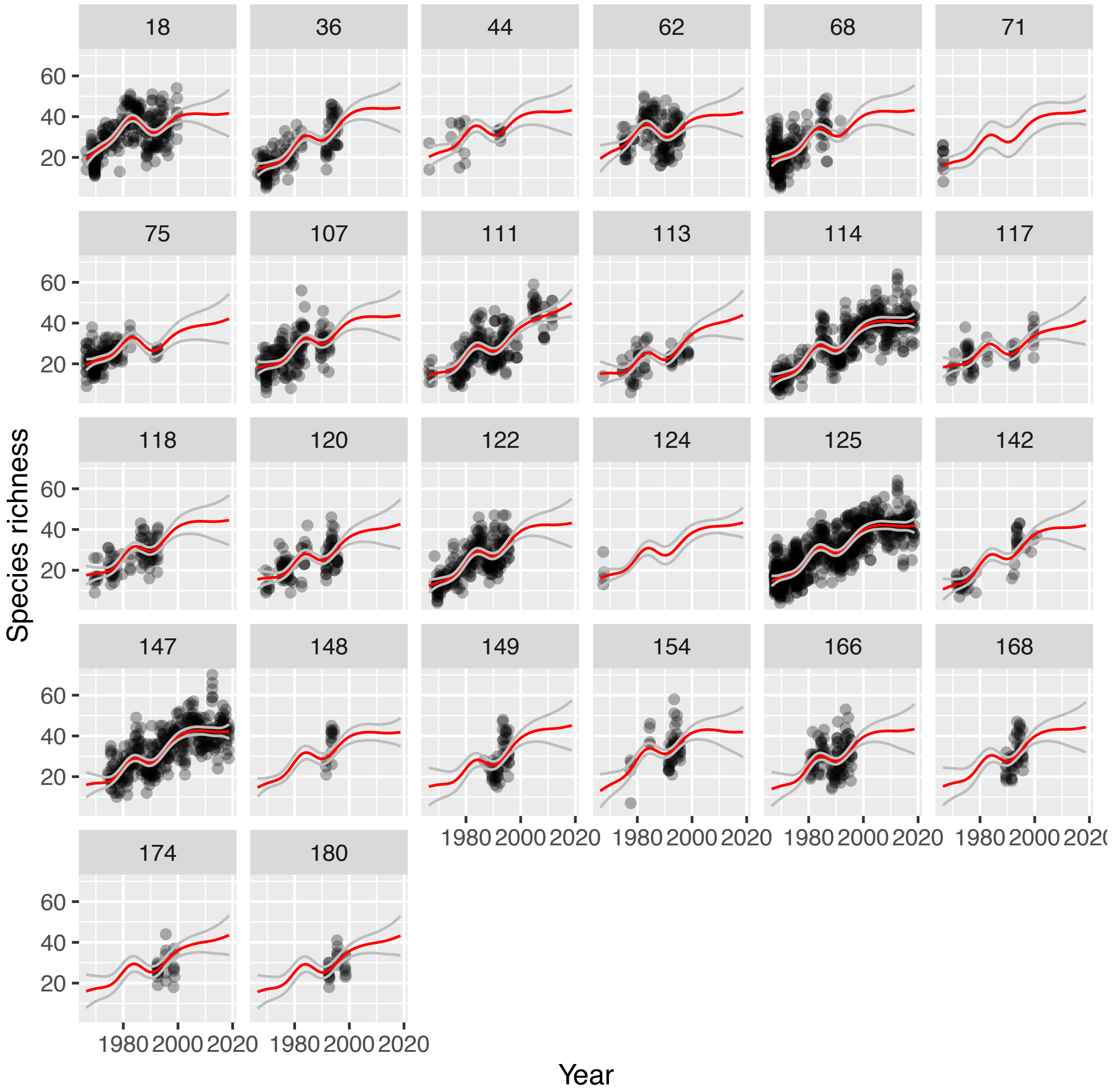

Figure S5. Hierarchical additive model fit of species richness against time in outer archipelago stations stations. The model has a common intercept and smooth term, but allows variation in the intercept and shape of smooth for each station (model GS). Station specific departures from the common intercept and smooth term are penalized. The red line represents the model fit of the long term trend; grey lines represent two times standard error of the fit. Original data points are plotted in the background with transparency. The station labels follow Figure 1.

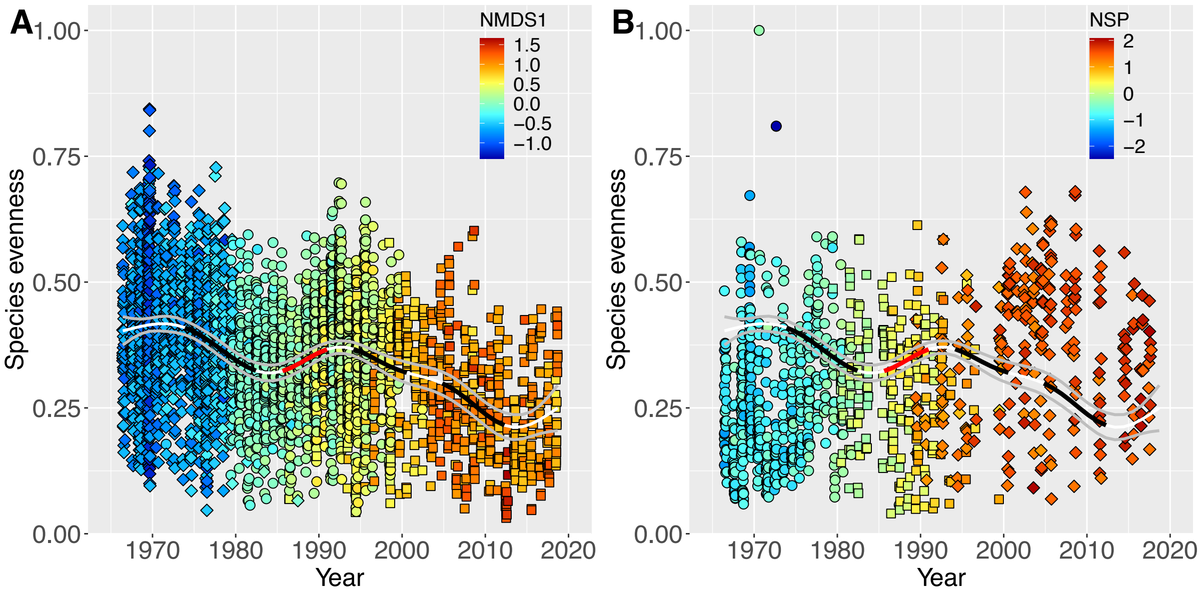

Figure S6. Decadal trends of phytoplankton species evenness in the pelagic sites (A) and inner bays (B). The symbol colour scales with NMDS1 scores and the shape corresponds to one of the three k-means cluster group. The GAM smooth lines of the year term (F = 41.4, edf = 7.7, n = 3515, R^2 = 0.19; F = 14.1, edf = 7.3, n = 1115, R^2 = 0.16 for A and B, respectively) are split into segments of significant ($p < 0.05$) increase (red), decrease (black) and no slope (white).

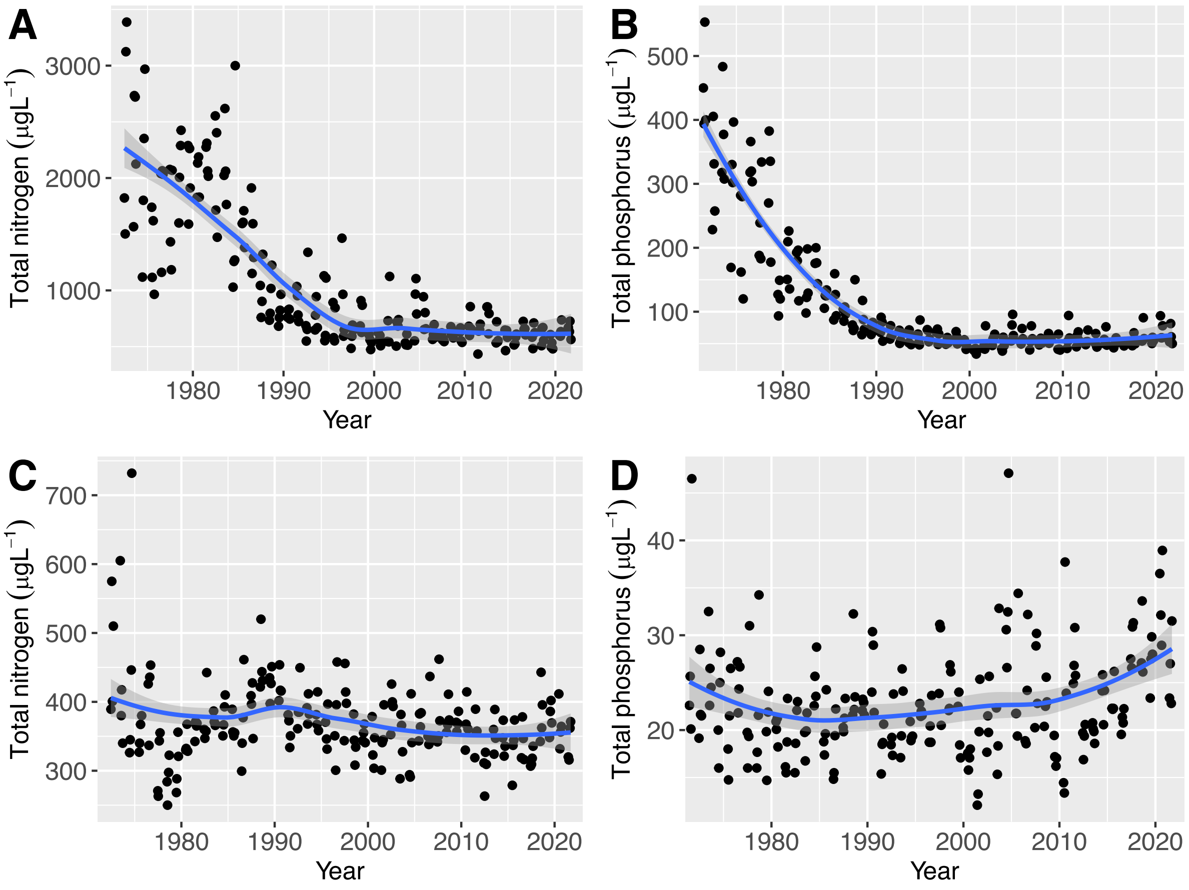

Figure S7. June to September monthly mean total nitrogen and phosphorus concentrations in the upper 20 m layer in the inner (A, B) and outer archipelago regions (D, D). The large decline in the inner bays was due to improved wastewater treatment. Total phosphorus shows recent increasing tendency in the outer archipelago, which is probably not related to wastewater treatment efficiency.

Table S1. Comparison of additive models depicting the time trends in phytoplankton community structure. For both, the outer archipelago stations and the inner bays, the response of three groups of variables against long term time trend were tested: scores of the first NMDS ordination axis (NMDS1), species richness (NSP), and species evenness (E10). The compared model types, with increasing complexity were: 0 – time and season as smooth terms, no autocorrelation or hierarchical structure; C – as 0, with added CAR(1) autocorrelation structure; hierarchical additive models: G – as C, but with added hierarchical structure, allowing a random effect of sampling station (intercept); GS – as G, but allowing for different shapes of smooths per station; GI – as GS, but also allowing for different wiggliness of the station specific smooths. The nomenclature of hierarchical models follows Pedersen et al (Pedersen *et al.*, 2019). Models were compared with Akaike's Information Criterion (AIC, the smaller the better), root means square error (RMSE, the smaller the better), and coefficient of determination (R^2). Anova-based hypothesis testing significance levels are as follows: **** p<0.0001, ** p < 0.001, * p> 0.05, or not significant compared to the previous model (one line above within a table block).

Outer archipelago				Inner bays			
model	AIC	RMSE	R^2	model	AIC	RMSE	R^2
NMDS1 0	-2036	10.58	0.914	NMDS1 0	106	8.109	0.937
NMDS1 C***	-3022	10.59	0.914	NMDS1 C***	35	8.21	0.937
NMDS1 G***	-3051	10.51	0.915	NMDS1 G ***	-128	7.56	0.946
NMDS1 GS***	-3098	10.33	0.917	NMDS1 GS***	-184	7.29	0.949
NMDS1 GI***	-3121	10.32	0.918	NMDS1 GI	-147	7.29	0.949
NSP 0	23849	421.75	0.561	NSP 0	7318	209.72	0.614
NSP C***	22889	422.18	0.560	NSP C***	7256	209.83	0.614
NSP G***	22278	397.13	0.608	NSP G***	7202	203.72	0.634
NSP GS***	22163	389.14	0.620	NSP GS***	7102	194.22	0.664
NSP GI***	22147	389.57	0.621	NSP GI**	7090	194.1	0.666
E10 0	-4932	7	0.186	E10 0	-1381	4.22	0.158
E10 C***	-5377	7.01	0.185	E10 C***	-1421	4.23	0.157
E10 G***	-5496	6.86	0.215	E10 G***	-1478	4.11	0.198
E10 GS***	-5515	6.81	0.224	E10 GS***	-1633	3.78	0.317
E10 GI	-5488	6.81	0.226	E10 GI	-1621	3.77	0.323

Table S2. Species richness of phytoplankton major taxonomic classes with occurrence optima in three sequential time periods. The time periods were chosen to include equal number of species, which totalled 114 per period in the outer archipelago and 98 species in the inner bays. The differences between these totals and the sums in the table correspond to a variety of smaller algal classes, which is not shown. Note that diatoms and Chlorophyceae tend to loose, while Trebouxiophyceae and dinoflagellates tend to gain species richness.

	Outer archipelago			Inner bays		
Time period	1	2	3	1	2	3
Time period range	1967-1988	1988-2002	2002-2018	1967-1985	1985-2000	2000-2019
Bacillariophyceae	52	31	15	37	30	10
Chlorophyceae	20	14	6	15	20	12
Trebouxiophyceae	7	8	15	8	7	14
Cyanophyceae	17	20	30	23	8	28
Dinophyceae	5	18	18	6	9	10
Sum	101	88	84	89	74	74

Table S3. Most frequent taxa of the major phytoplankton classes, which have their occurrence optima (centre of gravity of likelihood of presence along the time axis) within the time periods specified in Table S2. Note that the frequency, as reported here, is the absolute number of samples, where the taxon was recorded. The total number of samples were 3515 in the outer archipelago and 1115 in the inner bays. Species falling into group 1 tend to have their occurrence biased towards early years, species in group 3 their occurrence biased toward later years, and species in group 2 have high occurrence likelihood in the middle of the period or occurred relatively evenly throughout the 52 years period.

		Outer archipelago		Inner bays	
Group	Class	Species	Frequency		Frequency
1	Bacillariophyceae	*Thalassiosira guillardii*	1158	*Thalassiosira guillardii*	637
1		*Diatoma tenuis*	837	*Nitzschia acicularis*	512
1		*Cyclotella glomerata f. glomerata*	655	*Cyclotella glomerata f. glomerata*	380
2		*Skeletonema marinoi*	1932	*Skeletonema marinoi*	419
2		*Chaetoceros wighamii*	1701	*Chaetoceros*	405
2		*Thalassiosira baltica*	1116	*Nitzschia longissima*	274
3		*Nitzschia longissima*	801	*Rhizosolenia minima*	103
3		*Rhizosolenia minima*	407	*Cyclotella choctawhatcheeana*	103
3		*Fragilaria*	406	*Chaetoceros tenuissimus*	92
1	Chlorophyceae	*Monoraphidium komarkovae*	591	*Monoraphidium komarkovae*	650
1		*Scenedesmus quadricauda*	526	*Tetradesmus obliquus*	575
1		*Raphidocelis danubiana*	476	*Scenedesmus quadricauda*	493
2		*Monoraphidium contortum*	3117	*Monoraphidium contortum*	1092
2		*Monoraphidium minutum*	423	*Chlamydomonas*	406
2		*Desmodesmus bicellularis*	297	*Monoraphidium minutum*	256
3		*Chlamydomonas*	871	*Scenedesmus*	102

3		*Kirchneriella*	478	*Raphidocelis sigmoidea*	69
3		*Scenedesmus*	89	*Phacotus*	62
1	Cyanophyceae	*Pseudanabaena limnetica*	1309	*Pseudanabaena limnetica*	674
1		*Aphanocapsa reinboldii*	1208	*Planktothrix agardhii*	647
1		*Planktothrix agardhii*	802	*Aphanocapsa reinboldii*	459
2		*Aphanizomenon flosaquae*	2364	*Merismopedia warmingiana*	757
2		*Merismopedia warmingiana*	1368	*Merismopedia tranquilla*	173
2		*Woronichinia compacta*	1090	*Aphanizomenon flosaquae*	155
3		*Aphanizomenon*	1199	*Anabaena*	183
3		*Anabaena*	694	*Aphanizomenon*	124
3		*Dolichospermum lemmermannii*	589	*Snowella atomus*	111
1	Dinophyceae	*Borghiella pascheri*	1161	*Borghiella pascheri*	257
1		*Protoperidinium*	247	*Protoperidinium*	77
1		*Glenodinium paululum*	110	*Amphidinium*	43
2		*Dinophysis acuminata*	2001	*Glenodinium*	263
2		*Glenodinium*	1314	*Oblea rotunda*	140
2		*Oblea rotunda*	995	*Katodinium*	119
3		*Gymnodinium*	979	*Kryptoperidinium triquetrum*	158
3		*Kryptoperidinium triquetrum*	792	*Heterocapsa rotundata*	129
3		*Protoperidinium brevipes*	730	*Dinophysis acuminata*	120
1	Trebouxiophyceae	*Oocystis borgei*	1511	*Oocystis borgei*	424
1		*Mucidosphaerium pulchellum*	394	*Actinastrum hantzschii*	376
1		*Actinastrum hantzschii*	145	*Mucidosphaerium pulchellum*	322
2		*Oocystis lacustris*	632	*Lagerheimia genevensis*	95
2		*Chlorella chlorelloides*	105	*Lagerheimia*	77
2		*Lagerheimia*	54	*Chlorella chlorelloides*	75
3		*Oocystis*	506	*Oocystis*	238
3		*Koliella spiculiformis*	220	*Koliella spiculiformis*	214
3		*Koliella*	159	*Choricystis*	122
